# The use of fluorescent Nile red and BODIPY for lipid measurement in microalgae

**DOI:** 10.1186/s13068-015-0220-4

**Published:** 2015-03-12

**Authors:** Judith Rumin, Hubert Bonnefond, Bruno Saint-Jean, Catherine Rouxel, Antoine Sciandra, Olivier Bernard, Jean-Paul Cadoret, Gaël Bougaran

**Affiliations:** IFREMER, PBA, 44311 Nantes, France; CNRS-UMPC, LOV UMR 7093, 06230 Villefranche-sur-mer, France; INRIA BIOCORE, 06902 Sophia Antipolis, Cedex France

**Keywords:** Nile red, BODIPY 505/515, Microalgae, Neutral lipid, Fluorescence, Biodiesel

## Abstract

Microalgae are currently emerging as one of the most promising alternative sources for the next generation of food, feed, cosmetics and renewable energy in the form of biofuel. Microalgae constitute a diverse group of microorganisms with advantages like fast and efficient growth. In addition, they do not compete for arable land and offer very high lipid yield potential. Major challenges for the development of this resource are to select lipid-rich strains using high-throughput staining for neutral lipid content in microalgae species. For this purpose, the fluorescent dyes most commonly used to quantify lipids are Nile red and BODIPY 505/515. Their fluorescent staining for lipids offers a rapid and inexpensive analysis tool to measure neutral lipid content, avoiding time-consuming and costly gravimetric analysis. This review collates and presents recent advances in algal lipid staining and focuses on Nile red and BODIPY 505/515 staining characteristics. The available literature addresses the limitations of fluorescent dyes under certain conditions, such as spectral properties, dye concentrations, cell concentrations, temperature and incubation duration. Moreover, the overall conclusion of the present review study gives limitations on the use of fluorochrome for screening of lipid-rich microalgae species and suggests improved protocols for staining recalcitrant microalgae and recommendations for the staining quantification.

**Electronic supplementary material:**

The online version of this article (doi:10.1186/s13068-015-0220-4) contains supplementary material, which is available to authorized users.

## Introduction

Microalgae are currently considered as potential actors for the third biofuel generation for several reasons. They can be produced, in a autotrophic manner and on non-agricultural land, be cultivated in seawater or wastewater and offer a higher lipid productivity than first-generation biofuels [1,2]. Reviews on the topic ‘microalgae and energy’ are flourishing to support this promising path [3-8]. Publications on new algae, manuscripts revisiting known algae and teams discovering new paths have become increasingly common. The main bottlenecks of the full chain process have been identified, with claims to contribute to the decrease of the environmental and economic costs of microalgal biofuel [9-12]. However, one of the cornerstones of working with lipid-producing microalgae that has barely been discussed is the necessity to carry out accurate, easy, reliable and repeatable measurements of lipid quantity and quality. This is especially true when triacylglycerols (TAG) are targeted as the neutral lipid storage form in the cell, as these represent a precursor that can be transformed into biodiesel via a transesterification step.

Some reliable techniques exist that require heavy and expensive equipment and take up working time of qualified technicians [13]: in addition to gravimetry measurements [14,15], a large panel of chromatography strategies have been developed, like thin-layer chromatography (TLC), high-pressure liquid chromatography (HPLC) and gas chromatography (GC) coupled with mass spectrometry (MS) [16]. With such an approach, the full process for a single TAG measurement takes a few hours, with a total cost (including equipment, manpower and consumables) above $50 to $100 per sample.

Fluorescent dyes offer an indirect measurement for lipids. They are easier and cheaper to use than the above methods and require a much smaller amount of equipment. These dyes bind specifically to the compounds of interest and have the additional advantage of offering a quick response [17,18]. Vital stains for the detection of intracellular lipid by fluorescence microscopy, spectrofluorometry or flow cytometry [19] need to be used. The most common dyes for lipids are Nile red [19] and BODIPY 505/515 [20]. Nile red and BODIPY 505/515 offer several advantageous characteristics for *in situ* screening [21]. The initial reason for using these fluorescent dyes was to perform fast screening of potential oleaginous microalgae in order to identify promising sources for commercial biofuel production [1,22]. Nile red was previously used in semi-quantitative techniques [18,23,24] but, with improved methods, is now widely used to quantify lipid level. BODIPY 505/515 has been used more recently as a potential alternative to Nile red. In fact, these dyes do not stain all microalgae successfully, even though methods are constantly improving, as reported in the literature. There is a real need to screen a large number of microalgae using a rapid, accurate and reliable method for detection and quantification of lipids produced [23,25], but caution must be taken concerning the sensitivity of fluorimetric methods and, in particular, the issue of fading (fluorescence extinction). This review provides an updated state of the art on fluorescent dyes and their use in the literature, with a particular focus on Nile red and BODIPY 505/515, which are the two most popular stains used to assess lipid content especially the neutral lipid content of microalgae.

### Lipid-staining fluorochromes: Nile red and BODIPY 505/515

Among markers that correlate fluorescence with the lipid content in microalgae cells, Nile red (9-diethylamino-5H-benzo[a]phenoxazine-5-one) is the most commonly used lipophilic stain for intracellular TAG detection in microalgae prior to BODIPY 505/515 (4,4-difluoro-1,3,5,7-tetramethyl-4-bora-3a,4a-diaza-s-indacene) which is a green lipophilic fluorescent dye recently used as an alternative to Nile red staining (Table 1).

**Table 1:** **Characteristics of fluorescent neutral lipid BODIPY 505**/**515 and Nile red used in microalgae**

**Name**	**BODIPY 505**/**515**	**Nile red**
**Chemical formula**	C_13_H_15_BF_2_N_2_	C_20_H_18_N_2_O_2_
**Molecular weight**	248.0817	318.37
**Target molecule**	Lipid droplets	TAG
**Usual wavelengths** **(Ex** **/** **Em)**	488/510	488-530/575-580

Excitation (Ex) and emission (Em) wavelengths are approximate values depending on measuring devices and literature data.

In the early nineties, Nile blue was introduced by Smith [26] as a histochemical stain for distinguishing between blue-stained neutral lipid and red-stained acid lipid. From then on, it was extensively used in histochemical and physiological studies [27-29]. Later, Nile red was synthesized from Nile blue oxidation and was mainly used as a vital and fluorescent stain for medical and embryological research purposes [30-32]. Nile red is a hydrophobic and metachromatic dye with poor solubility and fluorescence in water, with colour emission varying from deep red to strong yellow gold in hydrophobic environments. Depending on excitation and emission wavelength, the dye has been used to stain different hydrophobic molecules. For instance, using the excitation/emission wavelengths 450 to 500/>528, Nile red can dye the cholesterol in the human plasma through staining of lipid vesicles in smooth muscle cells and in cultured macrophages incubated at low density [33,34]. It was also used to study membrane heterogeneity [35] and ligand-hydrophobic protein surface interactions with the alternative wavelengths 570/610 [36] and to study enzyme mechanism by using the wavelengths 550/640 to 660 [37]. Nile red has also been successfully used to stain intracellular neutral lipids that is, TAG and cholesterol esters in yeast, fungi with coupled wavelengths 488/565 to 585 [38] and also in microalgae, with wavelengths set to 488 to 525/570 to 600 [19] or to stain total lipids with wavelengths set to 490/585 [18]. Under some conditions, a high correlation between neutral lipid content, as measured by gravimetric method, and Nile red fluorescence was observed for a variety of microalgae, such as *Chlorella* sp. [24,39], *Crypthecodiniium cohnii* [17], *Tetraselmis suecica* [40], *Nannochloropsis gaditana* [41] and *Isochrysis affinis galbana* [42] but also with polar lipid concerning *Crypthecodiniium cohnii* [17] and *Tetraselmis suecica* [40] (Table 2).

**Table 2:** **Correlations for lipid measurement obtained with Nile red or BODIPY fluorescence intensity and gravimetrical determination**

**Class**	**Order**	**Species**	**NR**	**BODIPY**	**Treatment**	**Correlation**	**Reference**
Cyanophyceae	Synechococcales	*Synechococcus* sp.	-	X	-	-	[43]
Bacillariophyceae	Naviculales	*Phaeodactylum tricornutum*	X	-	-	-	[44]
			-	X	Ethanol	-	[44]
Chlorophyceae	Chlamydomonadales	*Chlamydomonas reinhardtii*	X	X	Glycerol/DMSO	-	[45]
		*Chlamydomonas* sp.	-	X	-	-	[46]
		*Dunaliella primolecta*	X	-	DMSO	-	[25]
		*Dunaliella salina*	X	-	-	*R* ^2^ = 0.90 (FL2/FL3)	[47]
		*Dunaliella tertiolecta*	-	X	Glycerol/DMSO	-	[48]
	Sphaeropleales	*Scenedesmus dimorphus*	X	-	-	*R* ^2^ = 0.9912	[49]
		*Scenedesmus obliquus*	X	-	-	-	[50]
		*Scenedesmus* sp.	-	X	-	-	[51]
			X	-	-	*R* ^2^ = 0.99	[52]
			X	-	Ultrasonic processor	-	[53]
		*Desmodesmus* sp.	X	-		*R* ^2^ = 0.972	[54]
Coccolithophyceae	Prymnesiales	*Chrysochromulina* sp.	-	X	-	-	[20]
Coscinodiscophyceae	Chaetocerotales	*Chaetoceros calcitrans*	-	X	-	-	[25]
Eustigmatophyceae	Eustigmatales	*Nannochloropsis oculata*	X	-	Glutaraldehyde	*R* = 0.856	[55]
			-	X	Glycerol/DMSO	-	[48]
		*Nannochloris atomus*	-	X	Glycerol/DMSO	-	[48]
		*Nannochloropsis gaditana*	X	-	-	Linearly correlated	[41]
		*Nannochloropsis* sp.	X	-	-	-	[23]
		*Crypthecodinium cohnii*	X	-	Glycerol/DMSO	-	[56]
			X	-	Algal powder	-	[57]
			X	-	Glycerol	-	[58]
			X	-	Glycerol	-	[59]
			X	-	-	*R* ^2^ = 0.9336	[17]
Peridinea	Peridiniida	*Tetraselmis* sp.	-	X	-	-	[43]
Prasinophyceae	Chlorodendrales	*Tetraselmis suecica*	-	X	Glycerol/DMSO	-	[48]
		*Tetraselmis suecica*	X	-	-	*R* ^2^ = 0.81	[60]
		*Tetraselmis subcordiformis*	X		DMSO	-	[44]
			-	X	Ethanol	-	[44]
			X	-	-	*R* ^2^ = 0.87	[40]
			-	X	-	*R* ^2^ = 0.93	[61]
		*Isochrysis affinis galbana*	X	-	-	*R* = 0.99	[42]
Prymnesiophyceae	Isochrysidales	*Isochrysis galbana*	X	-	Glutaraldehyde	*R* = 0.804	[55]
		*Isochrysis galbana*	X	-	-	*R* = 0.83 (FL2/FL3)	[62]
		*Isochrysis* sp.	X	-	-	*R* ^2^ = 0.93	[63]
		*Mallomonas splendens*	-	X	-	-	[20]
Synurophyceae	Synurales	*Auxenochlorella protothecoides*	X	-	Ethanol	*R* ^2^ = 0.888	[64]
Trebouxiophyceae	Chlorellales	*Auxenochlorella protothecoides*	-	X	-	-	[64]
		*Chlorella pyrenoidosa*	X	-	-	*R* = 0.83 (FL2/FL3)	[40]
		*Chlorella saccharophila*	X	-	Lyophilization	*R* ^2^ = 0.9706	[65]
		*Chlorella sorokiniana*	X	-	-	-	[50]
		*Chlorella* sp.	X	-	Lyophilization	*R* ^2^ = 0.99	[24]
			X	-	Algal powder	-	[57]
		*Chlorella vulgaris*	-	X	-	*R* ^2^ = 0.95	[49]
			X	-	Electric field	-	[66]
			X	-	DMSO	-	[39]
			X	-	DMSO	-	[25]
	Trebouxiales	*Botryococcus braunii*	X	-	-	*R* ^2^ = 0.997	[18]
Xanthophyceae	Mischococcales	*Ophiocytium maius Naegeli*	-	X	-	-	[20]

Determination and correlation coefficients obtained with Nile red or BODIPY 505/515 staining are expressed with the associated microalgae species and treatment. The cross indicates which dye was used.

BODIPY or boron dipyrromethene is a class of strong ultraviolet-absorbing molecules demonstrating a relatively sharp emission peak. These dyes are rather insensitive to pH and the polarity of their environment. It is possible to fine tune their fluorescence characteristics by making only small modifications to their structure, resulting in a variety of dyes with different excitation and emission maxima [49,67]. As such, these fluorochromes are widely used to label protein, DNA [67], fatty acids, phospholipids, cholesterol, cholesteryl esters and ceramides [68-70]. BODIPY 505/515 has been previously used to stain lipid-containing yolk platelets in living zebrafish embryos [71] and lipid-containing vesicles in immortalized human hepatocytes [72]. More recently, BODIPY 505/515 was reported to successfully stain lipid vesicles in microalgae [20,25]. Moreover, a good correlation was observed for *Tetraselmis subcordiformi*s lipid vesicle measurements between BODIPY 505/515 fluorescence and gravimetric analysis [61].

### Spectral properties and solvents

#### Spectral properties

Nile red used as an *in situ* marker provides high fluorescence in hydrophobic environments [32-34,73]. However, the spectral properties of Nile red are highly sensitive to the polarity of the immediate environment [74]. The peak emission is blueshifted as the surrounding polarity decreases [19,23,75,76]. The polarity dependence of Nile red may be attributed to large changes in the excited state dipole moment of the molecule. Indeed, twisted intramolecular charge transfer (TICT) processes are induced by the flexible diethylamino end group attached to the rigid structure of these molecules [76]. Emission fluorescence shifts can also result from binding of Nile red with certain proteins containing a hydrophobic domain (very low-density proteins) and other non-lipid cellular compartments containing a hydrophobic domain [34,49,77,78]. Therefore, Nile red does not specifically bind to lipid droplets and these properties may bias the lipid determination with this dye. Additionally, high pigment concentration may also interfere with lipid-induced Nile red fluorescence. Indeed, quantification of the carotenoid content of cells is possible using Nile red, thereby demonstrating the existence of a link between these two compounds [79]. Moreover, the high content of chlorophyll (1% to 4% of dry weight) found in some classes of microalgae, such as *Chlorophyceae*, is another source of interference and increase in the background fluorescence. Together with the use of particular wavelengths, a high pigment content could therefore prevent reliable lipid quantification with Nile red [39,80]. Nevertheless, the metachromatic properties of Nile red induced by microenvironment polarity have been exploited by some authors [17,40,47,62,81] to estimate the polar lipid/neutral lipid ratio (Table 3). Indeed, short excitation wavelengths (450 to 500 nm) and yellow/gold emission (≤580 nm) wavelengths favour the detection of highly hydrophobic environments like neutral lipids (TAG) whereas longer excitation wavelengths (515 to 560 nm) and red emission (≥590 nm) wavelengths favour a general fluorescence for polar lipids resulting from interactions with intracellular membrane phospholipids [34].

**Table 3:** **Correlation of PUFA**, **unsaturation index**, **neutral or polar lipid measurement obtained with Nile red fluorescence intensity and gravimetrical quantification**

**Class**	**Order**	**Species**	**PUFA content**	**Fatty acid unsaturation index**	**Neutral lipid**	**Polar lipid**	**Reference**
Chlorophyceae	Chlamydomonadales	*Dunaliella salina*	*R* = 0.83	*R* = 0.84	-	-	[62]
Prasinophyceae	Chlorodendrales	*Tetraselmis suecica*					
Prymnesiophyceae	Isochrysiales	*Isochrysis galbana*					
Trebouxiophyceae	Chlorellales	*Chlorella pyrenoidosa*					
Chlorophyceae	Chlamydomonadales	*Dunaliella salina*	*R* ^2^ = 0.90	*R* ^2^ = 0.83	-	-	[40]
Peridinea	Peridiniida	*Crypthecodinium cohnii*	-	-	*R* ^2^ = 0.93	*R* ^2^ = 0.83	[17]
Prasinophyceae	Chlorodendrales	*Tetraselmis suecita*	*R* ^2^ = 0.83	-	*R* ^2^ = 0.87	*R* ^2^ = 0.61	[41]

Correlation or determination coefficients of PUFA content and fatty acid unsaturated index were estimated between gravimetric measurements and the FL3/FL2 ratio measurement of Nile red fluorescence for microalgae species or group of algae species. Determination coefficient of neutral and polar lipids was obtained with gravimetric quantification and Nile red staining.

Unlike Nile red, BODIPY 505/515 is insensitive to the environment polarity [49] and numerous studies have established its specificity for lipid droplets. When excited with a blue laser (450 to 490 nm), BODIPY 505/515 gives a green peak emission ranging from 515 to 530 nm [25,48,49]. According to Cooper *et al*., (2010) [20], BODIPY 505/515 can be used to visualize lipid droplets within microalgae cells and the fluorochrome permeates all structures within a cell. The characteristic green fluorescence occurs when the dye reaches the lipid droplets [25,61,64]. Indeed, BODIPY 505/515 has the advantage of not binding to cytoplasmic compartments other than lipid bodies and chloroplasts [21], and the red autofluorescence from algal chloroplasts under the same blue excitation wavelength is spectrally distinct from the green fluorescence of the lipid droplets [20,48].

### Solvents

To improve staining by Nile red and BODIPY 505/515 dyes, a variety of solvents such as acetone, dimethylsulfoxide (DMSO), ethanol, dimethylformamide (DMF), isopropanol, ethylene glycol, hexane or chloroform have been used as stain carriers [19,33,34,73,75,76,82].

Fluorescent compounds such as Nile red, which contain polar substituents like polar carboxyl function (−COOH) on the aromatic rings, demonstrate emission spectrum sensitivity to the chemical and physical properties of the solvents [83], including: (i) variations in maximum fluorescence; (ii) alterations in the shape of the spectral curves, because of specific solvent effects such as hydrogen bonding or some other interactions [34]; and (iii) quenched fluorescence in aqueous media, resulting in a blueshift of the fluorescence maximum with decreasing solvent polarity, being 632 nm for ethanol, 600 nm for chloroform and 576 nm for hexane [21,23,34]. Nile red is more commonly diluted in acetone than in DMSO or isopropanol (Table 4). Apart from its polarity sensitivity, volatility of the solvent may also alter the fluorescence measurements. Although not mentioned in the literature, preservation of the Nile red solution may cause problems. Indeed, when acetone was used as the solvent, the repeated use of a given Nile red solution over a long period resulted in changes in the solution concentration [84].

**Table 4:** **Solvents used for Nile red solutions and the microalgae species on which they were applied**

**Class**	**Order**	**Species**			**Solvent**		
			*Acetone*	*DMSO*	*Isopropanol*	*Ethanol*	*DMF*
Bacillariophyceae	Naviculales	*Phaeodactylum tricornutum*	[52]				
	Bacillariales	*Cylindrotheca* sp.	[85]				
		*Nitzschia* sp.	[86]				
	Naviculales	*Amphiprora* sp.	[86]				
Chlorodendrophyceae	Chlorodendrales	*Tetraselmis* sp.	[23]				
Chlorophyceae	Chlamydomonadales	*Dunaliella primolecta*	[25]				
		*Dunaliella salina*	[40]				[84]
		*Dunaliella tertiolecta*		[51]			
	Sphaeropleales	*Scenedesmus dimorphus*	[87]			[82]	
		*Scenedesmus* sp.	[52,53]				
		*Neochloris oleoabundans*	[88]				
		*Scenedesmus obliquus*	[46]			[82]	
		*Ankistrodesmus pseudobraunii*		[39]			
Coscinodiscophycea	Thalassiosirales	*Skeletonema marinoi*	[89]				
	Chaetocerotales	*Chaetoceros calcitrans*	[25]				
		*Chaetoceros socialis*	[89]				
Dinophyceae	Dinotrichales	*Crypthecodinium cohnii*	[17]				
		*Gymnodinium* sp.	[86]				
	Gonyaulacales	*Alexandrium minutum*	[89]				
Eustigmatophyceae	Eustigmatales	*Nannochloropsis* sp.	[23,57,90]				
		*Nannochloropsis gaditana*	[42]	[42]			
Prymnesiophyceae	Isochrysidales	*Tisochrysis lutea*	[49]				
Trebouxiophyceae	Chlorellales	*Auxenochlorella protothecoides*				[82]	
		*Chlorella pyrenoidosa*	[57]				
		*Chlorella saccharophila*			[65]		
		*Chlorella sorokiniana*	[46]				
		*Chlorella* sp.		[24]			
		*Chlorella vulgaris*	[25,57]	[39]		[82]	
		*Chlorella zofingiensis*	[87]	[39]			
		*Parachlorella kessleri*			[50]		
		*Pseudochlorococcum* sp.	[87]				
	Trebouxiales	*Botryococcus braunii*	[18]				

DMF, dimethylformamide; DMSO, dimethylsulfoxide.

BODIPY 505/515 solution is most often prepared in DMSO, as this is thought to be more efficient for diffusion through cell membranes [20] (Table 5). Few studies have assessed the effect of solvents on BODIPY 505/515 fluorescence in microalgae. A decrease in fluorescence and a higher amount of cellular debris were reported when using high acetone and DMSO concentrations [49]. This study highlighted that the use of 1% to 2% acetone ensured cell integrity and a high fluorescence signal. Conversely, even low DMSO concentrations reduced BODIPY 505/515 fluorescence and dramatically increased the cell debris. These observations led [49] to the conclusion that organic solvents should be kept to a minimum.

**Table 5:** **Solvents used for BODIPY 505**/**515 solutions and the microalgae species on which they were applied**

**Class**	**Order**	**Species**			**Solvent**		
			*Acetone*	*DMF*	*DMSO*	*Ethanol*	*Isopropanol*
Bacillariophyceae	Naviculales	*Phaeodactylum tricornutum*				[44]	
Chlorodendrophyceae	Chlorodendrales	*Tetraselmis suecica*			[64]		
		*Tetraselmis subcordiformis*			[73]		
		*Tetraselmis* sp.			[91]		
Chlorophyceae	Chlamydomonadales	*Chlamydomonas* sp.			[92]		
		*Dunaliella primolecta*	[25]		[25]		
		*Dunaliella teteriolecta*			[64]		
	Sphaeropleales	*Scenedesmus dimorphus*	[67]		[67]		
		*Scenedesmus* sp.			[43]		
Coccolithophyceae	Prymnesiales	*Chrysochromulina* sp.			[20]		
Coscinodiscophycea	Chaetocerotales	*Chaetoceros calcitrans*	[25]		[25]		
Eustigmatophyceae	Eustigmatales	*Nannochloropsis oculata*			[64]		
		*Nannochloropsis* sp.			[91]		
Synurophyceae	Synurales	*Mallomonas splendens*			[20]		
Trebouxiophyceae	Chlorellales	*Auxenochlorella protothecoides*		[82]			
		*Chlorella vulgaris*	[25,67]		[25,67]		
		*Nannochloris atomus*			[64]		
Xanthophyceae	Mischococcales	*Ophiocytium maius*			[20]		

DMF, dimethylformamide; DMSO, dimethylsulfoxide.

As for Nile red, the preservation of BODIPY 505/515 solutions is little documented, but it is recommended that it should be stored in a dark bottle protected from light [25].

### Permeation issues and improvements

#### Dye permeation into microalgae

Nile red incorporation into microalgae is a sequential transfer of the dye from the plasma membrane to the lipid droplets. *In vitro* studies on isolated plasma membranes and isolated lipid droplets from *Dunaliella salina* compared to *in vivo* studies highlighted a clear biphasic fluorescence rise [84]. A first step of fast insertion/dissociation of the dye into and from the plasma membrane (half time of 1 to 2 s) was followed by a slower step where transfer from the plasma membrane to the lipid droplets occurred (half time of 30 s to 2 min). Indeed, interactions of the dye with proteins and/or other cellular components in cytosolic cells are major delaying factors. Fluorescence and staining kinetics depend on the microalgae species and the size of lipid droplets as well as on the amount of the latter. The localization of Nile red in lipid droplets is not clearly defined, but it is hypothesized that Nile red molecules could be embedded deep in the lipid core of the droplets [84].

Conversely, BODIPY 505/515 incorporation is very fast due to its high oil/water partition coefficient that allows the dye to cross cell and organelle membranes easily [20]. Using the ‘non-stop flow’ method [87], which allows continuous data gathering before and after dye addition, fast permeation into different algal cells (*Nannochloropis oculata*, *Nannochloropsis atomus*, *Dunaliella tertiolecta* and *Tetraselmis suecica*) was observed, resulting in the attainment of a fluorescence maximum within 1 min [48].

#### Permeation issues

The first limitation to staining with lipophilic dyes is related to the composition and structure of the algal cell wall. The robust and thick wall particularly widespread among green algae is thought to act as a barrier preventing Nile red dye from efficiently penetrating cells and staining lipids [21,39,56,62,84,90,93]. A poor correlation between Nile red staining and gravimetric methods was reported [39] in *Chlorella vulgaris* and *Pseudochlorococcum* sp. No difference for Nile red fluorescence was reported between the late stationary and the exponential phase in *Nannochloropsis* [56], whereas the gravimetric method revealed that the content doubled between these phases. This study showed that only 25% of the cell population was successfully stained when using the conventional Nile red staining method [56].

In parallel, BODIPY 505/515 seems to be more efficient for permeation. Recent studies showed that it penetrates all microalgae cells, even those with thick cell wall. Among a variety of algal taxa that store oil, *Ophiocytiummaius Naegeli* (Xanthophyceae), an elongated cell, *Chrysochromulina* sp. (Haptophyceae), a naked microalga, and *Mallomonas splendens* (Synurophyceae), an alga covered with elaborated silica scales, all allow the dye to penetrate [20]. Working with *Chlorella vulgaris*, *Dunaliella primolecta* and *Chaetoceros calcitrans*, Govender *et al*. (2012) reported, in agreement with Cooper *et al*. (2010), that BODIPY 505/515 was able to label lipid vesicles of these algal cells, which are known to have a thick, robust wall [20,25]. However, a recent study reported a low stain permeation rate across the thick and rigid cell wall of *Nannochloropsis oculata* cells [48], demonstrating low fluorescence in this species and a differentiation between fully and partially labelled cells.

#### Improved permeation protocols for staining recalcitrant microalgae

Several authors report failure of efficient cell staining with Nile red, caused by the difficulty of penetrating the cell wall, resulting in a poor fluorescence signal [21,39,84,90,93,94]. Some studies aimed to develop new protocols that improve dye penetration and ultimate staining of lipid droplets. Several chemical treatments have been proposed, such as DMSO, ethanol, acetone, ethylene glycol, isopropanol and glutaraldehyde, at various concentrations [39]. Other physical treatments, such as grinding of algal cells in liquid nitrogen, were also tested; these avoid the use of a solvent or detergent that could lead to cell damage, destruction of lipid globules or high background fluorescence [84].

#### Chemical treatments

*DMSO*. The polar organic solvent DMSO (dimethysulfoxide) is well known for its interaction capabilities with the cellular membrane and its cryoprotectant properties. DMSO facilitates permeation of macromolecules, fluorescent lipids and conjugated fluorescent dyes into live cells and tissues. Use of DMSO as a solvent for Nile red [24,39,41,50,95] and BODIPY 505/515 [20,21,25,43,46,48,49,51,61,64] makes it possible to exploit this permeation property. Its mechanism could be the induction of water pores across the lipid bilayer, together with modification of the membrane fluidity [92]. DMSO was proposed as a means to improve Nile red staining permeability in organisms like yeast [93] and microalgae mixtures [25,39,56,90,91] where it was added in the range from 5% to 20% depending on studies. A final DMSO concentration of 5% was used with *Pseudochlorococcum* sp. and *Scenedesmus dimorphus* [90], 15% was used with *Nannochloropsis* sp. [56] and 20% with mixed green algal cells [91]. Some studies show a maximum fluorescence efficiency at a DMSO concentration of 25% (v/v) in *Chlorella vulgaris* [39]. Below and above this concentration, the fluorescence efficiency decreased [39].

The disadvantage of the 20% DMSO method is that high DMSO concentrations affect cell survival and cannot be applied *in vivo* [84]. Indeed, growth inhibition of *Nannochloropsis* sp. exposed to DMSO concentration varying from 0.077 to 0.11 g mL^−1^ (7% to 10%, respectively) was reported; and culture exposed to 0.165 g mL^−1^ (15%) even collapsed after re-inoculation into fresh f/2 medium, thus demonstrating a toxic effect of DMSO [56].

*Glycerol*. The microalgae plasma membrane is permeable to small uncharged polar molecules like glycerol. Diffusion of certain molecules can be facilitated by adding glycerol to the extracellular medium. Indeed, the transport of Nile red across cell membranes of *Nannochloropsis* sp. was facilitated by glycerol at 0.05 g mL^−1^ [56,58]. The maximized efficiency of intracellular lipid staining was achieved by adding glycerol to the *Nannochloropsis* sp. suspension to a final concentration of 0.1 g mL^−1^ (with a Nile red incubation of 5 min at room temperature in darkness and a Nile red concentration of 0.3 μg mL^−1^): fluorescence intensity with glycerol treatment was sixfold higher than without glycerol. However, Pick and Rachutin-Zalogin (2012) [84] reported that glycerol quenched Nile red fluorescence when the dye was in excess (high Nile red/TO ratio).

Glycerol was also used together with BODIPY 505/515 at a similar concentration to Nile red. In this case, maximum fluorescence intensity was reached for a glycerol concentration of 0.1 g mL^−1^ [48] with *Nannochlopsis oculata*.

Interestingly, glycerol has no known cell growth inhibition effects, even when cells experience a high concentration of 0.125 g mL^−1^ for 1 h [56]. This property is an advantage for a staining procedure with flow cytometry cell sorting.

#### Physical treatments

The use of chemical treatment does not successfully allow the determination of TAG content in all green microalgae strains. Therefore, physical treatments were tested on recalcitrant microalgae species impermeable to lipid dyes.

#### Microwave-assisted staining

Chen *et al*. (2011) [90] suggested the use of additional treatments, such as the combination of DMSO treatment with microwave irradiation (e.g., on *Pseudochlorococcum* sp. ASU strain 1, *Scenedesmus dimorphus* ASU strain 1). Microwave irradiation increases molecular collision and movement speeds between algal cells and dye molecules and facilitates the penetration of the dye into cells [90]. Testing several microwave durations [90], the microwave-assisted staining resulted in the determination of a lipid content comparable to that obtained with the conventional gravimetric method for the screening of oleaginous algae (*Pseudochlorococcum* sp. *Scenedesmus dimorphus* and *Chlorella zofingiensis*). This demonstrates the reliability of this protocol *in vivo*. However, reliability and reproducibility issues were reported due to the uneven distribution of radiation within microwave ovens (particularly household microwave ovens) and the use of replicates was thus recommended [90].

#### Electric field

Molecule transport into microalgae intracellular compartments could be enhanced and accelerated by the establishment of an electric field [96]. Su *et al*. (2012) [66] tested three electric field intensities (0; 500; 1,000; and 2,000 V cm^−1^) on Nile red staining with *Chlorella vulgaris* and *Spirulina* sp. Applying the electric field for 10 s after Nile red addition to the electroporation chamber resulted in higher fluorescence intensity and lower variability.

#### Microalgae lyophilization

An improvement of Nile red staining was obtained for seven *Chlorella* strains using lyophilized algae as the raw material [24]. This technique eliminates the negative effect of the environment on fluorescence staining. A high correlation coefficient was found between spectrofluorometry quantification and the conventional gravimetric method [24].

### Staining parameters

A reliable estimate of the neutral lipid content in microalgae with dye fluorescence depends upon a variety of staining and measuring conditions. The use of non-optimal or inconstant conditions often results in poor lipid content assessment. Additionally, in most cases, the staining and measuring procedure is highly species specific.

#### Algae and dye concentrations

Since fluorescence alterations can arise from the cell and dye concentration: the quenching issue (that is, a non-radiative relaxation of excited electrons to the basic state) is well documented and can result in a dramatic fluorescence loss that prevents reliable estimation of the lipid content. Quenching is related to a variety of processes including excited-state reactions, energy transfer, dimerization between aromatic cycles of molecules and collisional quenching [34,36,83].

#### Algal concentration

A fluorescence decrease related to microalgae cell concentration was shown below a minimum threshold and above a maximum threshold of cell concentration for lipid staining, where saturation occurs. A linear correlation was established between microalgae concentration and fluorescence intensity between these thresholds. However, the optimum range of cell concentration is species specific and varies between 5.10^4^ and 1.10^6^ cell mL^−1^, as demonstrated by the results with *Chlorella vulgaris* [39,65], *Dunaliella salina* (personal communications), *Scenedesmus dimorphus* [49] and *Isochrysis* sp. [63].

#### Dye concentration

Studies carried out with Nile red and BODIPY 505/515 showed that fluorescence intensity depended on concentrations of the dyes [24,39,61]. Fluorescence intensity increased and then decreased with rising dye concentration. Existence of an optimal concentration resulting in a maximum fluorescence was established to obtain optimal lipid staining for both dyes.

At low concentration of Nile red, the hydrophobic core could interact with the dye, leading to an emission peak at 570 nm [34]. Moreover, again at low Nile red concentration (0.5 μM), quenching was found to be quite minor compared with observations at high concentration (4 μM) [84]. Using an *in vitro* test with triolein (TO) as a standard of triacylglycerol, the authors indicated that, when Nile red concentration is low relative to lipid droplets (Nile red/TO), the dye was less accessible to hydrophilic quenchers than at an excess Nile red concentration (Nile red/TO) [84]. Nevertheless, with increasing concentrations, the excess Nile red could not react with only neutral lipids or could interact with the phospholipidic coat and hydrophobic protein surfaces [36]. It resulted an emission peak at λ = 650 nm [89] induced by a redshift of the emission peak, which interferes with the peak related to the dye bound to neutral lipids (580 nm). A concentration-dependent self-association of Nile red was also reported when the dye was added in excess [84], and aggregated dye precipitates were difficult to gate based on red channel fluorescence as the chlorophyll of microalgae using flow cytometry [67]. Nile red dimers and aggregates interfere with fluorescence intensity of neutral lipids because of radiative or non-radiative energy transfer [97]. Special attention must, therefore, be paid to the use of excess Nile red concentrations for staining neutral lipids.

The concentration of Nile red required for staining microalgae varies considerably (0.01 to 100 μg mL^−1^) between the different species [24,25,39].

According to Govender *et al*. (2012) [25] and Xu *et al*. (2013) [61], all the studied microalgae displayed a significant optimal value in fluorescence intensities related to the BODIPY 505/515 concentration used.

Below optimal BODIPY 505/515 concentration, the dye did not adequately stain all cells. Above the optimal concentration, over-staining and bright green background fluorescence were visible, making quantification difficult and inaccurate for some species [25]. Furthermore, like Nile red stain, aggregated dye precipitate were also observed using flow cytometry and interfering with red channel fluorescence of chlorophyll [49].

The optimal concentration for BODIPY was around 0.067 μg mL^−1^ for three species of microalgae belonging to the Trebouxiophyceae, Bacillariophyceae and Chlorophyceae classes (*Chlorella vulgaris*, *Dunaliella primolecta and Chaetoceros calcitrans*) at 1.10^6^ cell mL^−1^ [25], whereas it was higher, 0.28 μg mL^−1^ for *Tetraselmis subcordiformis* (Chlorodendrophyceae) at 1.10^6^ cell mL^−1^ [61].

### Temperature

Very few studies have investigated the effects of staining temperature at the time of labelling, although this is a factor that influences fluorescence: fluorescence responses subsequent to Nile red staining were studied between 20°C and 80°C for *Chlorella vulgaris*, and the most appropriate staining temperature range was found to be 37°C to 40°C [39]. The authors hypothesized that high temperature facilitated permeation of Nile red into cells. Most Nile red analyses are based on these staining temperatures for several microalgae classes: Eustigmatophyceae, Chlorophyceae, Trebouxiophyceae and Peridinea [17,41,47,50,57,60,64,91] whereas room temperature was used for other analyses with Eustigmatophyceae and Prymnesiophyceae classes [42,56,58,59]. These studies support the idea that staining temperature is not species dependent for Nile red.

Testing the effect of temperature in the range 20°C to 45°C, optimal incubation temperature for BODIPY 505/515 was found to be 25°C when staining *Nannochloropsis oculata* cells [48]. Most other studies with BODIPY 505/515 also used the same temperature for a variety of microalgae classes: Eustigmatophyceae, Chlorophyceae, Trebouxiophyceae and Coscinodiscophyceae [20,25,43,46,49,51,61,64].

### Salinity

Limitation of Nile red fluorescence was reported [84] under hypersaline condition for *Dunaliella salina*. A lower Nile red fluorescence, a shift of the peak emission and a retardation of the dye transfer into lipid droplets were observed with high salinity (0.5 to 3 M NaCl) [84]. Indeed, some species, such as *Dunaliella* sp., adapt to high salt concentration and counterbalance the external high osmolarity by massive accumulation of internal glycerol [98]. This reaction induces a fluorescence quenching of Nile red as demonstrated on artificial model systems, phospholipid vesicles and triolein vesicles [84]. It appears that salinity affects Nile red through another distinct process by strongly affecting the solubility of the solvent [99]. Therefore, Nile red miscibility in salted water changes with a change of salinity.

### Incubation duration

Intensity of Nile red fluorescence is not constant over time in microalgae [19,23,35,38,84]. The shape of the fluorescence curve is species specific [19,84]. After Nile red addition, fluorescence increases to reach a peak and then decreases, according to different slopes for *Dunaliella salina*, *Dunaliella parva*, *Dunaliella bardawil* and *Nanochloris atomus* [84], or reaches a plateau, for *Chlorella vulgaris* [39]. The fluorescence maximum is not reached at the same time for different species [23,84].

Several Nile red incubation times are proposed in the literature. A 5-min incubation time is used for *Nannochloropsis* sp. [56,58,59], whereas a 15-min incubation time is used with *Tetraselmis suecica* [60]. Nile red fluorescence was measured for incubation periods of between 30 s and 14 min with the diatom *Amphora coffeaeformis*, revealing that a maximum and stable fluorescence could be obtained with a treatment between 2 and 7 min [19]. Beyond this duration, fading (reduction of fluorescence intensity) took place in the samples. Similarly, variation in the maximum emission intensity was found with time and algal strain (*Nannochloropsis* sp. or *Tetraselmis* sp.), leading to the recommendation to use the largest fluorescence intensity signal following Nile red incubation lasting from 30 to 40 min [23]. A 10-min incubation period is largely used with Nile red in the literature [25,39,47,50,94]; some authors established that this Nile red incubation time is optimal to avoid fluorescence fading in *Chlorella vulgaris* and *Dunaliella salina* [39,84].

More precisely, it was established that photobleaching occurs with Nile red analyses as the fluorescence lifetime progressively decreases over time for *Dunaliella primolecta*, *Chlorella vulgaris* and *Chlorella calcitrans* [25].

Moreover, Nile red fluorescence quenching is species specific [84]. Pick and Rachutin-Zalogin (2012) pointed out that, after long incubation, Nile red fluorescence quenching occurred, resulting from interactions with cell compounds rather than the destruction of the dye from enzymatic degradation. In their study, fluorescence level decreased by only 4% to 6% in cell extracts after 15 h of incubation, whereas it decreased by 84% to 93% in intact cells.

BODIPY 505/515, in contrast, is insensitive to light and oxidation [25]. Maximum fluorescence value is reached within a minute after the incorporation of BODIPY 505/515 in the microalgae cultures of *Nannochloropsis oculata*, *Nannochloris atomus*, *Tetraselmis suecica* and *Dunaliella tertiolecta* [48]. Fluorescence intensity is maintained for a period as long as 11 days with *Nannochloropsis oculata*, *Nannochloris atomus*, *Tetraselmis suecica* and *Dunaliella tertiolecta*, suggesting that BODIPY 505/515 is also insensitive to photobleaching [48].

It is perhaps for this reason that some studies do not indicate the incubation period used. However, an optimal incubation period of 10 min was established for BODIPY 505/515 staining after testing durations from 2 to 25 min with *Tetraselmis subcordiformis* [61]; this timing has also been used for *Chlorella vulgaris*, *Dunaliella primolecta* and *Chaetoceros calcitrans* [25] and microalgae species of several other phyla [43].

According to Cooper *et al*. (2010) and Brennan *et al*. (2012) [20,48], the BODIPY 505/515 stained the intracellular oil-containing organelles within minutes and the subsequent photostability permitted confocal time-lapse measurements of the lipid bodies over a 20-min period.

In these studies, Nile red and BODIPY 505/515 were used in darkness and protected from the light [25].

### Blank measurements

#### Relative fluorescence, obtained after subtraction of a control measurement, is sensitive and requires an accurate blank measurement

When using Nile red staining, fluorescence related to medium impurities should be taken into account either with a washing step or the use of a blank. Bertozzini *et al*. (2011) performed cell centrifugation and resuspension in a freshly prepared medium in order to eliminate impurities [89]. The use of a blank with Nile red is complex. First, red autofluorescence from algal chloroplasts may interfere with Nile red fluorescence of lipid droplets and thus increase fluorescence background [39,80]. The dye fluorescence in the medium can also be significant, especially for old cultures, which may have excreted various molecules. When cultures have been stressed, they produce more extracellular polymeric substances and/or transparent exopolymeric particles (TEP). These molecules, mainly constituted of carbohydrates, proteins and some lipids, may react with Nile red. Indeed, macromolecules containing sugars (the major constituent of TEP) have a native fluorescence that can be measured with Nile red [100,101]. Thus, both intra- and extracellular compartments are concerned for blanks. In contrast to analysis with a fluorimeter, lipid droplet analysis with a flow cytometer makes it possible to eliminate the signal due to the extracellular compartment. In the literature, blanks are computed on the basis of i) non-stained samples in order to estimate the autofluorescence background of the medium and correct for cellular autofluorescence [17,49,52,62] or ii) the sum of the cell autofluorescence and the dye fluorescence in the medium [18,24,89].

Blanks are simpler with BODIPY 505/515. First, autofluorescence from chlorophyll *a* does not interfere with BODIPY 505/515 fluorescence. However, this dye produces a high fluorescence background even without cells. Over-staining and bright green background fluorescence can be visible and sometimes too high to allow quantification [20,25,64]. Quantification of lipid droplet fluorescence with BODIPY 505/515 using a microplate-based fluorometric method has not been documented [64]. Staining lipid droplets with BODIPY 505/515 is mainly performed by flow cytometer, which avoids the fluorescence background of the medium and explains the limited information given about blanks in the literature. However, non-stained samples can be used as a control [48] with cytometric analysis.

## Discussion

The present review summarizes the available knowledge for the two main fluorochromes commonly used for TAG measurement in microalgae: Nile red and BODIPY 505/515. We showed how preliminary studies on staining procedure are important and clearly depend on the scientific question being examined, in relation to the microalgae species concerned.

### Choosing between Nile red and BODIPY 505/515

For decades, Nile red has been extensively used to label lipid droplets in microalgae cells. Numerous studies have listed the difficulties occurring when using this dye with microalgae (that is, limitation of the labelling due to issues of permeation, conservation of the dye, fluorescence quenching and photostability). The major disadvantages of Nile red are its i) limited photostability, ii) interference with chlorophyll [80] and iii) difficulty of permeation for some species. Solutions have been proposed in the literature related to the labelling and the quantification of lipids. Optimal conditions for quantification of the lipid content are again specific to each microalgae species. However, many studies on Nile red staining demonstrated the effectiveness of this dye with microalgae species (Table 2).

BODIPY 505/515 was recently presented as a better marker than Nile red for visualizing neutral lipid content in fluorescence microscopy studies [20,64]. BODIPY 505/515 labelling of lipid bodies in live cells is clearly and distinctly seen with both wide-field epifluorescence microscopy and confocal microscopy [20]. Recent studies used BODIPY 505/515 for lipid droplet analysis, thus avoiding the use of Nile red. Nevertheless, some authors have reported disadvantages of BODIPY 505/515, such as i) background fluorescence of the dye in the medium and ii) failure to quantify neutral lipids between rich and low oil strains.

Currently, the comparison of Nile red and BODIPY 505/515 remains a complex question and it is difficult to show that the use of one or the other is better because the available data are rather difficult to compare. Nile red has been studied for many years as a probe for microalgae lipids and its mechanisms are starting to be understood, whereas BODIPY 505/515 is a new molecule for which many details are still lacking. Nevertheless, the marking response is clearly species dependent, which makes it possible to choose the most adapted dye for the microalgae species and tools available, as shown in Table 2. For both reagents, authors have suggested improvements for marker incorporation into microalgae cells. The efficiency of such enhanced methods is largely proven, even though certain Nile red and/or BODIPY 505/515 labelling still fails for some species. These species-specific technique improvements do however raise issues about i) the speed of the method and ii) possible errors caused by the toxicity of treatments to the cells [44].

### Applications of fluorochromes

#### Screening

Even if Nile red or BODIPY 505/515 staining remains a powerful quantification tool in terms of time and cost of biomass [21,64], high-throughput quantification methods of lipids with Nile red or BODIPY 505/515 fluorescence can hardly be seen as a method for screening different species of microalgae. It has been pointed out that staining protocol is species specific. Therefore, a given fluorescence value cannot be associated with the same amount of TAGs in two different species [64]. Thus, monitoring of oil-rich microalgae species is still possible for small samples when high frequency screening is required but not suitable for a large-scale analysis [57]. However, high-throughput quantification of lipids with fluorochromes is widely applicable for screening of oil-rich cells in intra-population studies for microalgae domestication and diversity analysis research programs [42,58,59].

#### Cytometry and cell sorting

High-throughput analysis based on flow cytometry analysis to perform rapid and quantitative processing of samples gives very good results both with Nile red [17,42,47,60,79] and, more recently, BODIPY 505/515 [20,25,48]. Relative to spectrometry, flow cytometry addresses the per-cell fluorescence and makes it possible to distinguish between cells, debris and precipitate fluorescence [49]. However, preparing samples for cytometry can be time consuming, particularly for small sample sets.

Analyses with flow cytometry allow fluorescent-activated cell sorting and selection of cells with specific characteristics [102]. It offers the possibility to isolate improved strains or transgenic algae strains [42,43,50,58-60]. Combined flow cytometry with microscopy (imaging flow cytometry using the Amnis ImageScream X) is a powerful tool to visualize and quantify high-throughput lipid droplets in microalgae species [103,104]. This tool provides visual lipid quantification responses with fluorochromes.

### Relative *vs* absolute quantitative measurement

The major drawback of fluorochrome techniques for TAG assessment is that absolute quantification cannot be addressed. To bypass this problem, some authors suggested the use of a lipid standard such as triolein (TO), and fluorescence measurements have since been referred to in TO equivalents. Triolein is a symmetrical triglyceride derived from glycerol and from three polyunsaturated oleic fatty acids. To validate this calibration, triolein quantity and fluorescence intensity measurement results must be linear and reproducible [95]. Several studies use a calibration curve with TO for absolute quantification of lipids [32,35,65,66,76,78,91,96]. Another approach for TAG measurement is also used and consists in the addition of different quantities of a standard to an algal culture to determine its lipid concentration [105]. Although this is a well-known technique in chemistry, it requires more investigation in the field of fluorescent markers [89]. Nevertheless, the use of the standard raises different problems. It is not easy to produce a stable, homogeneous and reproducible solution of triglycerides. Priscu *et al*. (1990) [86] noticed that correlation with fluorescence of cell lipids is not straightforward, which is due to fluorescence differences between staining of lipid standards in cell-free solution and staining of algal cells. The main reasons for these differences lie in hydrophobicity, size and numbers of micelles formed between agglomerated TAG standards and intracellular lipid bodies in the algal strains, which may result in possible overestimation or underestimation [64]. Moreover, the standard addition method through the use of TO is applicable to certain algal species such as *Bacillariophyceae* and *Dinophyceae* but not to algal strains with thick cell walls that prevent diffusion of the Nile red or BODIPY 505/515 into the lipid vesicles [89]. Alternatively, absolute quantitative measurements can be also made with a calibration curve obtained with a microalgae culture of different lipid concentrations.

### Recommendations for lipid quantification by fluorescent markers

Since staining with a fluorochrome dye is a very fast and cheap method to assess the lipid content in microalgae, it has attracted increasing attention over the last decade. Therefore, only a few studies propose a comprehensive procedure to perform Nile red [64,84] and BODIPY 505/515 staining on microalgae.

**Figure 1:**
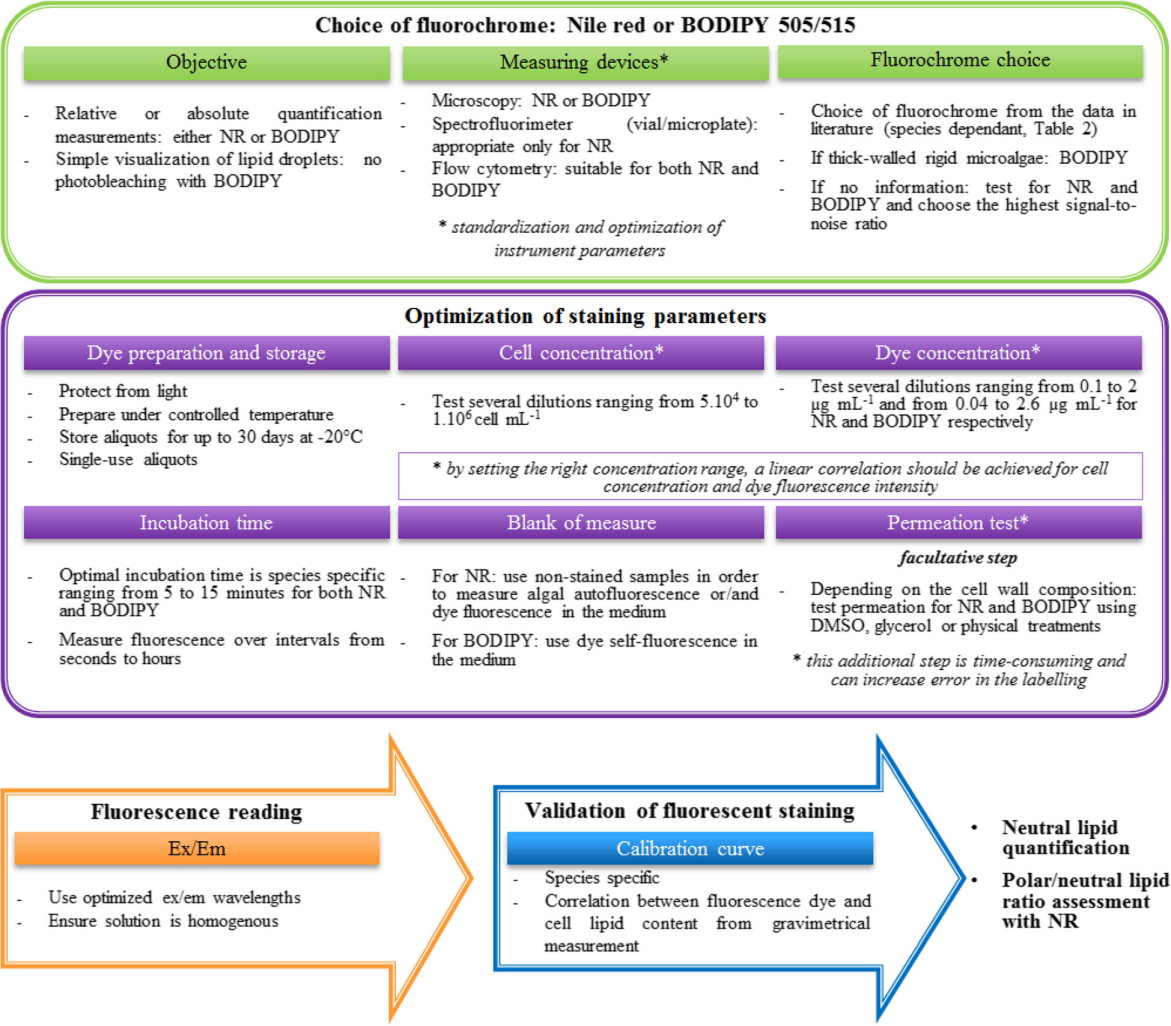
**General flow chart of an optimal protocol for lipid quantitative measurements in microalgae cells.** Setting of fluorescent measurement procedure exposed different steps from choice of fluorochrome to neutral lipid quantification with operational indication for both Nile red (NR) and BODIPY 505/515 (BODIPY). DMSO, dimethylsulfoxide; Em, emission; Ex, excitation.

The development of an adapted staining protocol is a long process to successfully define a reliable means of assessment of the microalgae lipid content. However, from the review of the existing literature, it is possible to provide a framework and guidelines that will be helpful for researchers aiming to use Nile red or BODIPY 505/515 in reliable conditions, with accuracy and reproducibility (Figure 1).

#### Objective and tools for the choice of fluorochrome

Evaluating the content of lipids in microalgae can be done by several alternative measurement approaches. First, fluorescence microscopy offers the possibility to calculate the volume of lipid vesicles [73]. This measurement can be carried out by staining with Nile red or BODIPY 505/515 but does not allow high-throughput study of microalgae lipid content. The use of a spectrofluorimeter with i) a classic vial or ii) a microplate allows faster measurements. The classic fluorimeter method in a vial requires a large amount of biomass (1 to 4 mL) and is relatively time consuming, as measurement is not automated. The use of a microplate allows a high-throughput method with several advantages in terms of time and does not require large amounts of biomass and solvent for the quantification of neutral lipid content. However, spectrofluorimeter analyses are more appropriate for Nile red staining because of the self-fluorescence of BODIPY 505/515 [44,83]. Then, flow cytometry allows efficient fluorescence measurement suitable for both Nile red and BODIPY 505/515. This high-throughput method also saves time and requires a lower algal biomass. In all cases, measurement sensitivity by fluorescence techniques requires standardization and optimization of instrument parameters [19,23]. Precision of staining conditions is essential for the reliability and robustness of the method.

Current studies show a good efficiency of Nile red staining for many species, but staining with BODIPY 505/515 seems more appropriate for thick-walled rigid microalgae. However, if no previous work is available for a particular species, tests of Nile red and BODIPY 505/515 analysis should be run separately. For each species, the fluorochrome that gives the highest signal-to-noise ratio should be chosen.

#### Optimization of staining parameters with Nile red and BODIPY 505/515

Dye stock solutions should be stored at controlled temperature and protected from the light, for up to 30 days at −20°C, avoiding thus fluorescence loss and evaporation. A safe option consists in preparing single-use aliquots of Nile red solution and storing these at low temperature and protected from light [84].

The optimal algal and dye concentrations are dependent on the microalgae species and can be determined by testing several dilutions. By setting the right concentration ranges, a linear correlation should be achieved for cell concentration and dye fluorescence intensity. Generally, the final concentration of Nile red in the culture sample ranges between 0.1 and 2 μg mL^−1^. In parallel, final concentration of BODIPY 505/515 ranges between 0.04 and 2.6 μg mL^−1^. The appropriate type of blank should then be used.

The kinetic of fluorescence intensity over time is species specific. Incubation times usually range between 5 and 15 min for both the Nile red and BODIPY 505/515. Fluorescence kinetics should be experimentally tested for each species in order to assess the optimal incubation. Fluorescence should be measured over intervals from seconds to hours, making sure that photobleaching does not occur between two measurements.

Permeation is a facultative and preliminary step that can be required depending on the cell wall composition. This step can be carried out using DMSO, glycerol or even physical treatments. However, since this is an additional time-consuming step which can moreover increase error in the labelling, its necessity must be carefully evaluated.

#### Fluorescence reading of fluorochromes

Fluorescence measurement to efficiently assess lipid quantity and quality in microalgae is carried out using optimized excitation and emission wavelengths. Using Nile red staining, information on lipid classes is obtained by the choice of the Ex/Em wavelength pair. These measurements allow information to be obtained on lipid composition of microalgae species via the polar/neutral ratio (Table 3). Care should be taken to ensure the homogeneity of the dye-culture mixture before reading the fluorescence.

#### Calibration of fluorescent staining

Lastly, for each species, a calibration curve would establish the relationship between fluorescence and cell lipids, as measured gravimetrically, and confirm the correct staining procedure (Table 2).

## Conclusion

This review highlights the key steps of a protocol for efficient lipid labelling in microalgae species with Nile red and BODIPY 505/515. It clearly appears that markers used without accurate preliminary work to optimize the protocol may result in quantification errors. The major advantage of lipid-droplet staining in microalgae cells using fluorochromes remains the possibility of high-throughput quantification. These fluorochromes are likely to become standard advanced biotechnology tools in the future and participate in a preliminary step of the scaling-up for the production of biofuels or feed oils. Additional means must be found in order to improve the high-throughput screening of microalgae candidates for various applications. Measurements of lipids per biomass unit (cell, carbon…) require a calibration curve correlating the fluorescence to lipid content, determined analytically. The key issue is that the calibration should be carried out regularly to demonstrate the relevance of protocol for the targeted species and its physiological state. Metabolic stress involving nutrient limitation [40,42,55,63,88,106,107] or enrichment [106] to enhance TAG content must be considered with caution, since the optimal staining protocol may not be the same in cells with different physiological states.

## Abbreviations

DMF: dimethylformamide
DMSO: dimethylsulfoxide
Em: emission
Ex: excitation
PUFA: polyunsaturated fatty acid
TAG: triacylglycerol
TO: triolein
